# Evaluation of the Effect of Distant Cold Stimulation on Pain During Palatal Injection: A Randomized Split-Mouth Study

**DOI:** 10.7759/cureus.37749

**Published:** 2023-04-18

**Authors:** Kinjal S Lakhani, Samir Joshi, Sudhir Pawar, Aishwarya U Lohokare, Priyanka B Rathod

**Affiliations:** 1 Oral and Maxillofacial Surgery, Bharati Vidyapeeth Dental College and Hospital, Pune, Pune, IND; 2 Oral Medicine and Radiology, Bharati Vidyapeeth Dental College and Hospital, Pune, Pune, IND; 3 Orthodontics and Dentofacial Orthopaedics, Bharati Vidyapeeth Dental College and Hospital, Pune, Pune, IND

**Keywords:** palatal injection, pain, local anesthesia, cold, cryotherapy

## Abstract

Objective

Delivery of a robust local anesthetic injection aids in the successful management of all patients' fears, anxieties, and discomfort during dental treatments. The most expected or frightening stimuli in the dental operatory are local anesthetic injections. The objective of this trial was to study the analgesic efficacy of distant cold stimulation in relieving injection pain from the greater palatine nerve block. Before receiving local anesthetic injections, employing cryotherapy by using an ice bath changes the pain perceptions and also increases the pain threshold.

Purpose

The aim of this study is to evaluate the effect of distant cold stimulation on palatal injection pain using an ice-cold bath.

Method

This was a randomized, controlled trial conducted at an oral and maxillofacial surgery department. A split-mouth technique was employed for the study, in which patients requiring bilateral greater palatine nerve block for any dental procedures were included. The bilateral greater palatine nerve block was given one at a time, separated by an interval of three days. The inclusion criteria for this study were no history of drug allergy and an extraction site free of any active infection. There were 28 participants in this experimental study. Two groups were randomly created from this research sample: group A (palatal injection with distant cold stimulation) and group B (palatal injection without distant cold stimulation). In group A, the patient was asked to put his or her hand of the same side as the palatal injection in an ice-cold bath till the time patient could tolerate it; immediately after the patient removed his hand, the greater palatine nerve block was given, and the patient was assessed for the injection pain. In group B, the patient was directly given the greater palatine nerve block without any distant cold stimulation. The time interval between the two extractions/dental procedures was three days. Outcomes of interest were pain severity with and without distant cold stimulation which were assessed using a Visual Analogue Scale (VAS) pain scale, and a comparison was made between the two groups.

Results

As per our study, in terms of pain, there was a statistically significant difference between the two interventions at all time points. Patients in group A had a lower score on the VAS pain scale as compared to group B. The standard deviation (STD) for group A was 0.81, and the standard deviation for group B was 0.92. P value was derived to be P < 0.001, which is suggestive of a significant difference between the pain scores of both groups.

Conclusion

Hence, we conclude that the use of distant cryotherapy as an adjunct is an effective way to reduce pain perception and increase pain threshold. This technique is comparatively simple, painless, and easy for the surgeon and for apprehensive patients, and it offers a fair cost solution for the suffering often associated with dental procedures requiring local anesthetic injections.

## Introduction

Local anesthetic injection to the palate in addition to buccal anesthesia is commonly used for dental treatments involving the maxillary posterior teeth. A dental phobia that lasts for an average of about 24 years might develop as a result of the terribly distressing experience that many patients have with palatal injections [1]. In this study, we assessed the pain after giving a greater palatine nerve block. The main branch of the palatine nerve that supplies the hard palate is called the greater palatine nerve. The greater palatine foramen allows it to enter the hard palate, where it travels in a groove almost to the incisor teeth and connects with the terminal filaments of the nasopalatine nerve there [2]. The patient's psychological state is negatively impacted by the site of injection or needle sensation. A proper dental procedure may be hampered by insufficient anesthesia, which increases the anxiety associated with needles [3].

To lessen the discomfort experienced during the administration of local anesthetic agents, especially during the palatal injection, researchers have employed a variety of desensitization techniques, including the use of topical anesthetics, warming and buffering of the anesthetic solution, counter-irritation, vibration or pressure, acupuncture, hypnosis, and computer-controlled delivery systems (WAND), utilizing several equipment such as dental vibe, vibrajects, or jet injectors to reduce discomfort experienced during the local anesthetic injection [4-9].

Another recommended method that has been shown to be efficient and cost-effective is the use of cryotherapy. In order to reduce discomfort from the injection site, ice is applied at another site; this alters the nerve conduction velocity [10]. The use of ice benefits the patient physiologically and psychologically, since it may divert their attention from the injection discomfort [11]. The nociceptive nerve fibers' speed of conduction is directly correlated with the lack of pain. The pace of nerve conduction is slowed down by cooling, which causes analgesia. In the spinal cord, nociception is prevented by cryotherapy by activating thermoreceptors, which contain temperature-sensitive nerve endings [12].

This study was undertaken for a comparative evaluation of the patient’s perceived pain response to the greater palatine nerve block which was given after exposure of the patient’s hand to an ice-cold bath for two minutes. This pain perception was assessed using a Heft-Parker Visual Analog Scale.

## Materials and methods

Study design

This was a single-center, prospective, randomized clinical trial comparing the local anesthetic injection pain with and without distant cold stimulation. The study was approved by our institutional review board and institutional ethics committee prior to inception. Informed written consent was obtained from all patients before carrying out the procedure.

Study setting and population

This study was conducted in an oral and maxillofacial surgery department. A convenience sample of 28 patients was identified by trained research assistants who received a briefing on the study criteria and objectives. These patients required bilateral extraction of teeth from the maxillary first premolar to the maxillary third molar. The subjects were selected keeping in mind the following inclusion criteria such as no history of drug allergy and extraction site free of any active infection. Patients were excluded from the study if they had a contraindication to the local anesthetic injection or had any prior allergy, medication intolerance, or history of peptic ulcer disease. This study was conducted by a split-mouth technique, wherein extractions were done one at a time separated by a gap of at least three days.

Study protocol and methodology

The collection of demographic and clinical data was done using a standardized data collection form. Data collected included demographic information such as the age and sex of the patient. Patients were given an information sheet and were explained in detail regarding the procedure and study. Written informed consent was taken from the patient before the procedure for publication of the study findings. A detailed case history was obtained from all the patients participating in the study, and standard sterilization protocol was followed throughout the study. All the patients were explained about the visual analog performa pre-operatively, which was filled by the patients based on their experience post local anesthetic injection. All injections and extractions were performed by the same surgeon, and all the parameters were recorded by the same clinician. Treatment assignments were enclosed in sequentially numbered, opaque, and sealed envelopes. The envelopes contained even proportions of the two study treatments. For each enrolled patient, the next in the series of envelopes was opened to reveal the treatment allocation.

**Table 1:** CONSORT Checklist CONSORT: Consolidated Standards of Reporting Trials, N/A: not applicable.

Section/Topic	Item No	Checklist item	Reported on Page No
Title and abstract			
	1a	Identification as a randomized trial in the title	Pg no. 1
	1b	Structured summary of trial design, methods, results, and conclusions (for specific guidance see CONSORT for abstracts)	Pg no. 1
Introduction			
Background and objectives	2a	Scientific background and explanation of the rationale	Pg no. 2
	2b	Specific objectives or hypotheses	Pg no. 2
Methods			
Trial design	3a	Description of trial design (such as parallel, factorial) including allocation ratio	Pg no. 3
	3b	Important changes to methods after trial commencement (such as eligibility criteria), with reasons	Pg no. 3
Participants	4a	Eligibility criteria for participants	Pg no. 3
	4b	Settings and locations where the data were collected	Pg no. 3
Interventions	5	The interventions for each group with sufficient details to allow replication, including how and when they were actually administered	Pg no. 3
Outcomes	6a	Completely defined pre-specified primary and secondary outcome measures, including how and when they were assessed	Pg no. 3
	6b	Any changes to trial outcomes after the trial commenced, with reasons	No changes
Sample size	7a	How sample size was determined	Pg no. 3
	7b	When applicable, explanation of any interim analyses and stopping guidelines	N/A
Randomization			
Sequence generation	8a	Method used to generate the random allocation sequence	Pg no. 3
	8b	Type of randomization; details of any restriction (such as blocking and block size)	Pg no. 3
Allocation concealment mechanism	9	Mechanism used to implement the random allocation sequence (such as sequentially numbered containers), describing any steps taken to conceal the sequence until interventions were assigned	
Implementation	10	Who generated the random allocation sequence, who enrolled participants, and who assigned participants to interventions	Pg no. 3
Blinding	11a	If done, who was blinded after assignment to interventions (for example, participants, care providers, those assessing outcomes) and how	N/A
	11b	If relevant, description of the similarity of interventions	N/A
Statistical methods	12a	Statistical methods used to compare groups for primary and secondary outcomes	Pg no. 6
	12b	Methods for additional analyses, such as subgroup analyses and adjusted analyses	Pg no. 6
Results			
Participant flow (a diagram is strongly recommended)	13a	For each group, the numbers of participants were randomly assigned, received intended treatment, and were analyzed for the primary outcome	Pg no. 5
	13b	For each group, losses and exclusions after randomization, together with reasons	Pg no. 3
Recruitment	14a	Dates defining the periods of recruitment and follow-up	
	14b	Why the trial ended or was stopped	
Baseline data	15	A table showing baseline demographic and clinical characteristics for each group	Pg no. 6
Numbers analyzed	16	For each group, the number of participants (denominator) included in each analysis and whether the analysis was by originally assigned groups	Pg no. 5
Outcomes and estimation	17a	For each primary and secondary outcome, results for each group, and the estimated effect size and its precision (such as 95% confidence interval)	Pg no. 5
	17b	For binary outcomes, the presentation of both absolute and relative effect sizes is recommended	N/A
Ancillary analyses	18	Results of any other analyses performed, including subgroup analyses and adjusted analyses, distinguishing pre-specified from exploratory	N/A
Harms	19	All important harms or unintended effects in each group (for specific guidance see CONSORT for harms)	N/A
Discussion			
Limitations	20	Trial limitations, addressing sources of potential bias, imprecision, and, if relevant, the multiplicity of analyses	Pg no. 7
Generalizability	21	Generalizability (external validity, applicability) of the trial findings	Pg no. 6
Interpretation	22	Interpretation consistent with results, balancing benefits and harms, and considering other relevant evidence	Pg no. 6
Other information			
Registration	23	Registration number and name of trial registry	7
Protocol	24	Where the full trial protocol can be accessed, if available	N/A
Funding	25	Sources of funding and other support (such as the supply of drugs), the role of funders	N/A

**Figure 1:**
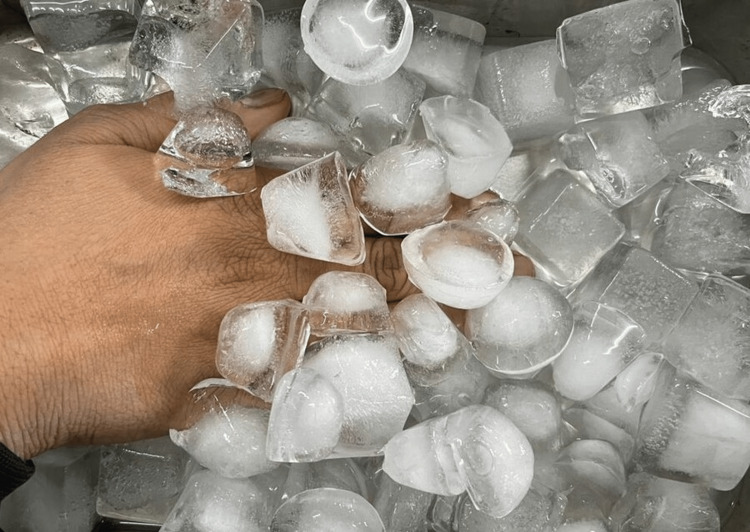
Ice Bath for Distant Cold Stimulation

Painting and draping were done under an aseptic protocol. An ice-cold bath was prepared by taking ice cubes in a steel tray. In the first appointment, the patient was asked to put his or her hand of the same side as the greater palatine nerve block injection in this ice-cold bath till the time patient could tolerate it (Figure 1). This was done for distant cold stimulation.

Immediately after the patient removed his hand, the greater palatine nerve block was given using 2% lignocaine with adrenaline and the patient was assessed for injection pain after the distant cold stimulation. The pain was evaluated immediately after the local anesthetic injection was given, using a 10-point Heft-Parker visual analog scale (VAS) marked by the patient herself or himself based on the pain experienced by the patient. Following this, a routine extraction procedure was carried out.

**Figure 2:**
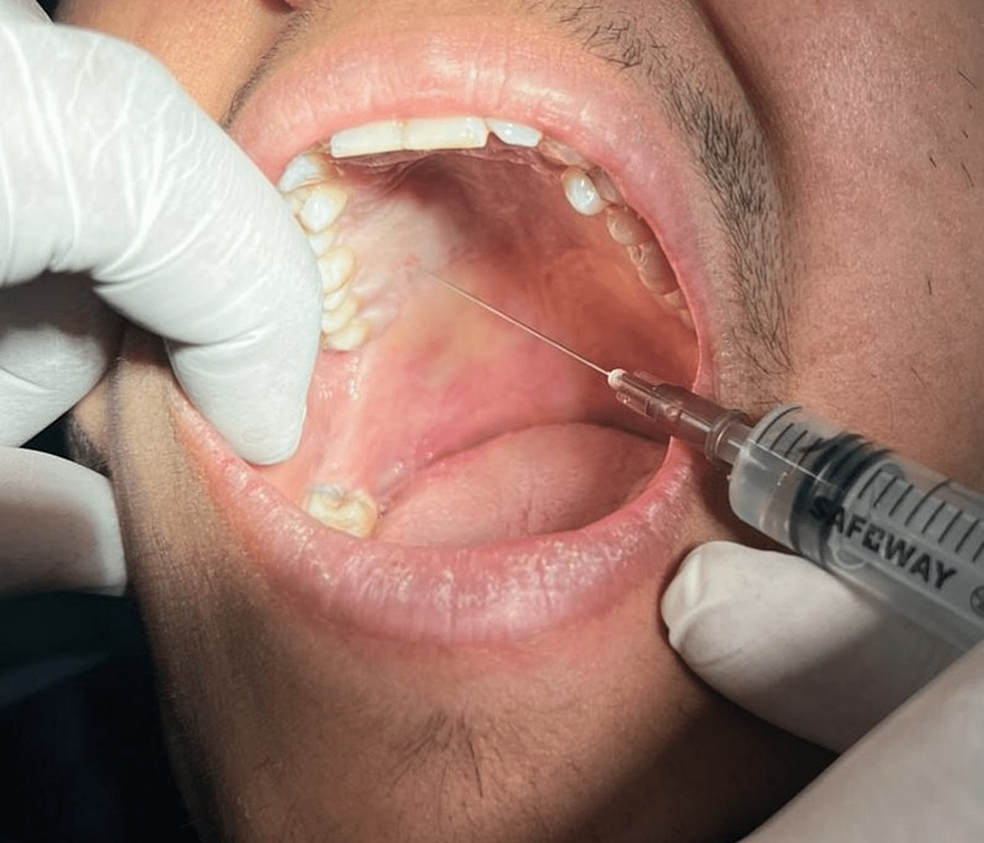
Greater Palatine Nerve Block Immediately After Cold Stimulation

For the extraction of the other side, the patient was directly given the greater palatine nerve block using 2% lignocaine with adrenaline, without any distant cold stimulation. Immediately following the local anesthetic injection, the patient was asked to mark the pain experienced during the injection on a 10-point visual analog scale. The time interval between the two extractions was three days. Due to the nature of the intervention, it was not possible to blind the patient and physician to the treatment allocation.

**Figure 3:**
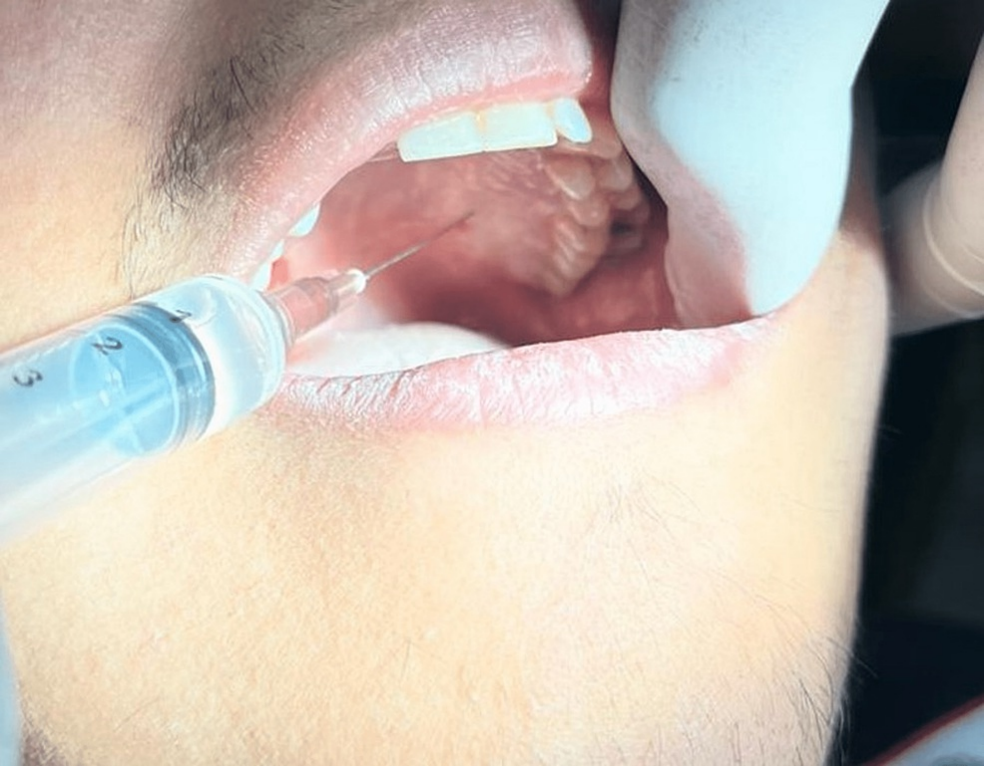
Greater Palatine Injection Without Cold Stimulation

Heft-Parker Visual Analogue Scale

It is a subjective method used to measure chronic and acute pain. It is a 10-pointer numeric rating scale on which patients rate their current pain intensity, ranging from “0” (no pain) to “10” (worst possible pain). These scores were recorded for all the patients and were then forwarded to the statistician for statistical analysis. All the patients were given post-operative instructions and medications following the extractions for three days. The patient was recalled after three days for the extraction in the opposite quadrant.

Outcome and measurements

The main outcome measure was the difference in pain severity score on a 10-point VAS pain scale with and without distant cold stimulation. VAS was marked by the patient immediately after the local anesthetic injection. The patient’s data were statistically analyzed using a software named IBM SPSS Statistics 20.0 (IBM Corporation, Armonk, NY, USA). All the statistical data were compiled in a tabulated and graphical format.

**Figure 4:**
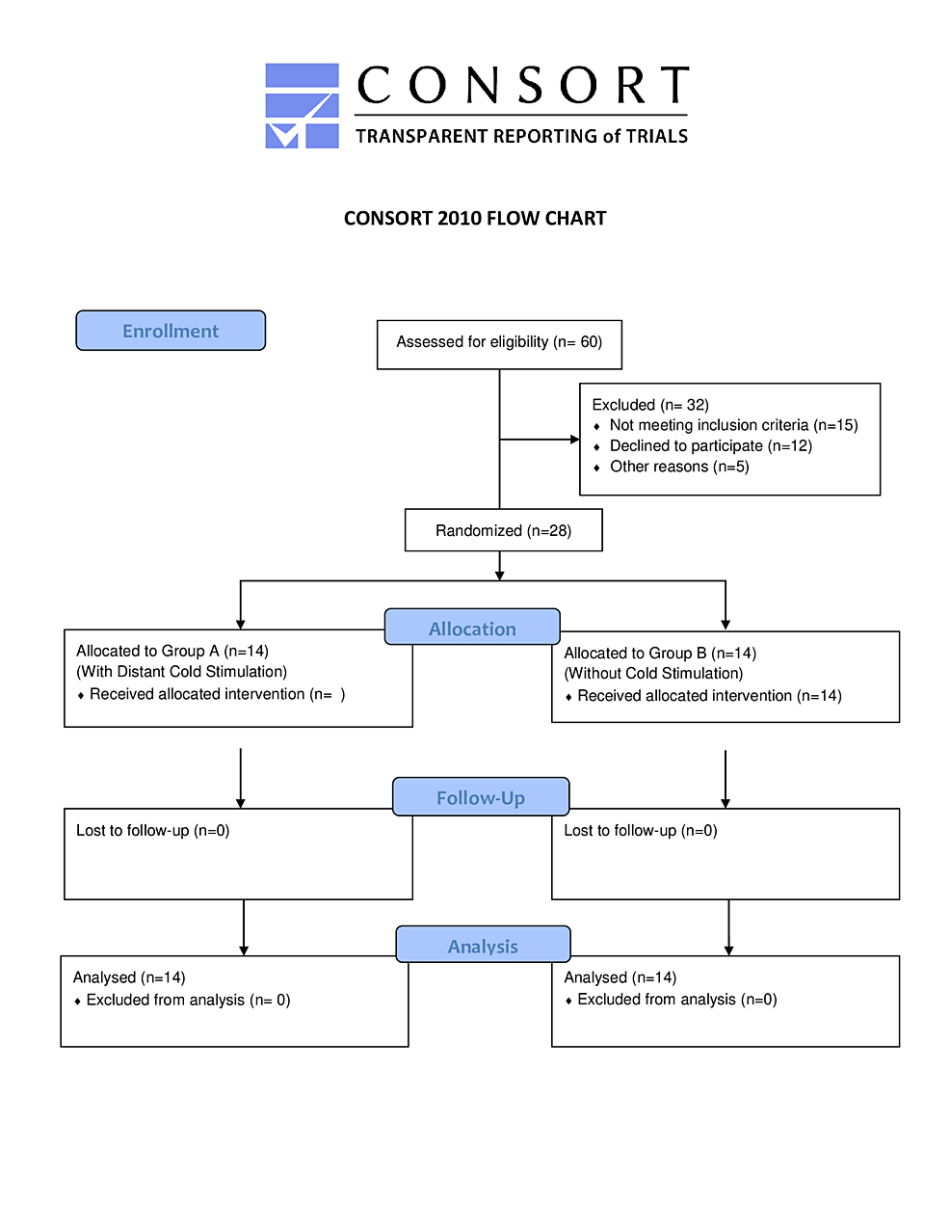
CONSORT Flow Chart CONSORT: Consolidated Standards of Reporting Trials.

## Results

**Figure 5:**
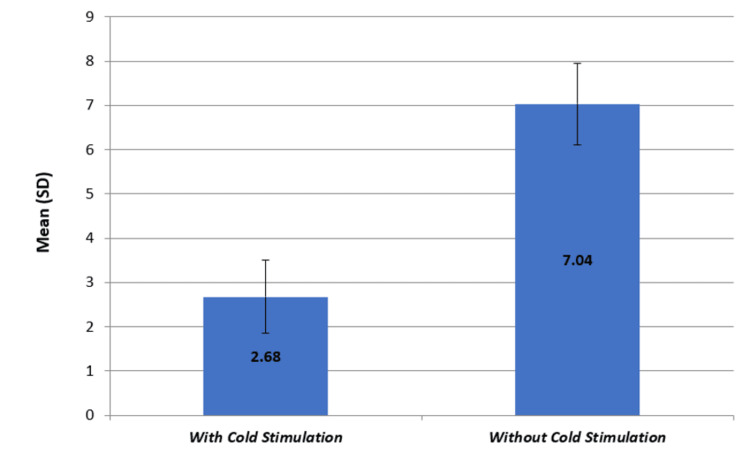
Comparison of the Pain Score With Cold Stimulation and Without Cold Stimulation (Graph)

Descriptive and inferential statistical analyses were carried out in the present study. Results on continuous measurements were presented on mean + SD. The level of significance was fixed at P = 0.05, and any value less than or equal to 0.05 was considered to be statistically significant. Student t-tests (two-tailed, unpaired) were used to find the significance of study parameters on a continuous scale between the two groups. The standard deviation for group A (with distant cold stimulation) was derived to be 0.81 and for group B (without distant cold stimulation) was derived to be 0.92 (Tables 2, 3). Here, the value of P derived was P < 0.001 which was inferred to be highly significant. The P value depicts that the use of distant cold stimulation is very effective to increase the pain threshold and decrease the intensity of injection pain (Figure 5). Groups were similar in baseline demographic and clinical characteristics.

**Table 2:** Descriptive Statistics

Patient	VAS Score (With Cold Stimulation)	VAS Score (Without Cold Stimulation)
1	2	6
2	2	6
3	3	7
4	2	6
5	3	8
6	4	8
7	2	7
8	2	7
9	2	6
10	3	6
11	2	9
12	3	8
13	5	9
14	3	6
15	2	7
16	3	7
17	3	8
18	2	7
19	3	7
20	2	8
21	2	6
22	4	6
23	3	7
24	2	6
25	2	7
26	2	7
27	4	8
28	3	7
Mean ± SD	2.68 ± 0.819	7.04 ± 0.922

**Table 3:** Comparison of the Pain Score in Terms of Mean (SD) Among Both the Groups Using Unpaired t Test P < 0.001: highly significant**.

Group	N	Mean	Standard Deviation	t Value	P Value
With cold stimulation	28	2.68	0.819	18.694	<0.001**
Without cold stimulation	28	7.04	0.922		

The statistical software IBM SPSS Statistics 20.0 (IBM Corporation, Armonk, NY, USA) was used for the analyses of the data, and Microsoft Word and Excel (One Microsoft Way, Redmond, Washington) were used to generate graphs, tables, etc.

## Discussion

Dental fear and needle phobia are the two biggest difficulties that a dentist faces in day-to-day life. Local anesthetic injections are an imperative part of all surgical procedures and dental extractions [13]. The systemic or local administration of cold for medicinal purposes is known as cryotherapy or cold treatment. Cryotherapy may be used to reduce discomfort caused by local anesthetic injections, and it also decreases the nerve conduction velocity [12]. The pain threshold is raised by cryotherapy via activating myelinated A-fibers and inhibitory pain pathways. Moreover, it slows down nerve conduction [14].

The "gate control theory of pain" may assist in comprehending the mechanism behind the analgesic action of cryotherapy. Cryotherapy when used, it acts as a defense against anesthetic injection. It reaches the brain before the pain experience does since the brain can only acknowledge one sensation at a time. Cryotherapy, a form of counterstimulation, thereby lessens pain perception. Although if the impact of cryotherapy lasts just a short while, it is sufficient to lessen the pain brought on by the insertion of an anesthetic needle [13]. Cryotherapy can be achieved by the application of an ice pack or by using an ice bath.

Many clinical studies have been carried out to evaluate the efficacy of cryotherapy in increasing the pain threshold. A clinical trial by Aminah et al. examined the effects of various desensitizing techniques on reducing injection pain in children, including local anesthetic gel, pre-cooling the injection site, vibration, and buffering the anesthetic solution, and came to the conclusion that cryotherapy significantly decreased the pain perception in children [15]. Similar studies were carried out by Duncan et al. and Hameed et al. who applied a refrigerant spray to the tissue before administering an intraoral injection, obtaining comparable results [16-17]. Aminabadi and Farahani discussed how well a two-minute cryotherapy treatment prior to nerve block reduced the pain perception and also increased the pain threshold [18]. The effectiveness of various pre-cooling agents (ice cone and refrigerant) and topical anesthetics (benzocaine) on pain perception during intraoral injection was evaluated by Garima et al. [19]. They found that the ice cone group had lower mean VAS ratings than the refrigerant and benzocaine groups. The authors indicated that ice may be more effective than refrigerant since it has more contact time with tissues [19]. Soni et al. compared the effects of lignocaine gel and pre-cooling for maxillary infiltrations among 50 children aged seven to 12 years using VAS and Structural Equation Modeling (SEM) measures for subjective and objective analyses. They said that the pre-cooling technique is a secure and reliable technique that also serves as a diversion when administering local anesthesia (LA) [20].

The purpose of the current study was to evaluate the effect of cryotherapy on perceptions of pain. This study has taken into account the palatal injection, which is thought to be more painful than other injections in the oral cavity. To avoid bias and take into account that each patient experiences pain differently, patients who needed bilateral greater palatine nerve block for any of the dental procedures were enrolled in this split-mouth study. The amount of pain experienced by the patient was recorded using a visual analog scale (VAS). The patient was instructed to place the hand of the same side as the palatal injection in an ice-cold bath for as long as they could tolerate it during their first appointment. As soon as they took their hand out, a greater palatine nerve block was administered, and the patient's tolerance for the injection pain was evaluated. The greater palatine nerve block was administered to the patient without any cold stimulation in preparation for the extraction of the opposite side. Three days passed in between the two extractions. The findings of this study confirm the hypothesis that cryotherapy greatly reduces pain at the time of needle penetration during local anesthetic administration for dental treatment. As compared to the control group, the scores on the VAS pain scale were lower in the cryotherapy group.

The outcomes of this study are consistent with the research done by Harbert, who used cold to reduce the pain perception associated with palatal injections [1]. This study proved that cryotherapy helps with patient management during dental treatments by increasing injection tolerance, while a local anesthetic is being delivered. The design of the present study could not control the confounding variable such as double blinding of the patients and depth of needle penetration while injecting local anesthesia. There is a negligible probability of a crossover effect in this split-mouth study as the techniques used in this study are completely mechanical. The distant cold application used in this study has no pharmacological effects on the body and hence does not require any wash-out period.

## Conclusions

Based on the results of this research, in terms of pain, there was a statistically significant difference between the two interventions at all time points which means that the pain scores in group A (with distant cold stimulation) were much less as compared to the pain score in group B (without cold stimulation). To conclude, cryotherapy administration prior to local anesthetic injection may be recommended as a simple and affordable auxiliary approach that is advantageous to all patients with fear and anxiety. It also lowers pain perception by acting as a defense against injection pain. It reaches the brain before the pain experience does since the brain can only acknowledge one sensation at a time. It raises the pain threshold via parasympathetic stimulation. It provides a reasonably priced remedy for the agony usually connected with dental procedures.
